# Pain and its association with device-measured activity patterns in older adults: age and sex differences in a population-based cross-sectional study

**DOI:** 10.1186/s11556-026-00422-0

**Published:** 2026-06-16

**Authors:** Caroline Lager, Abigail Dove, Debora Rizzuto, Joan Ars, Anne-Marie Boström, Amaia Calderón Larrañaga, Anna-Karin Welmer

**Affiliations:** 1 Division of Physiotherapy, Department of Neurobiology, Care Sciences and Society, Karolinska Institutet, Stockholm, Sweden; 2 Aging Research Center, Department of Neurobiology, Care Sciences and Society, Karolinska Institutet, Stockholm, Sweden; 3 RE-FiT Barcelona Research group, Vall d’Hebron Institute of Research and Parc Sanitari Pere Virgili, Barcelona, Spain; 4 Division of Nursing, Department of Neurobiology, Care Sciences and Society, Karolinska Institutet, Stockholm, Sweden; 5 R&D unit, Stockholms Sjukhem, Stockholm, Sweden; 6 Stockholm Gerontology Research Center, Stockholm, Sweden

**Keywords:** Physical Activity, Accelerometer, Movement behaviour, Older Adults, Pain, Latent Class Analysis

## Abstract

**Background:**

While pain has been widely studied in relation to physical activity (PA), less is known about how habitual patterns of activity relate to pain in older adults. This study examined associations between pain characteristics and activity patterns in older adults, and whether these associations differ by age and sex.

**Methods:**

Cross-sectional data from 665 older adults in the Swedish National Study on Aging and Care–Kungsholmen (SNAC-K, 2016–2019) were analysed. PA and sedentary behaviour were measured using the activPAL3™ accelerometer, and pain characteristics were collected via questionnaires. Latent Class Analysis identified three activity classes (named *Active-*,* Balanced and Sedentary Movers*) based on steps/day, sedentary time- and bout duration, light PA, moderate-to-vigorous PA, and number of light-/moderate-to-vigorous PA events. Multinomial logistic regression assessed associations between activity classes and pain, testing modification by age and sex.

**Results:**

In the total sample, higher pain intensity was associated with membership in the *Sedentary Movers* class (RRR: 2.16, 95% CI: 1.06, 4.40, *p* = 0.034), characterised by low PA and predominant sedentary behaviour, compared with the *Active Movers* (reference), characterised by high activity levels with substantial light- and moderate-to-vigorous PA. The interaction analysis displayed that associations were concentrated in women and the oldest-old (≥ 80 years). No significant associations were observed for pain and the *Balanced Movers* vs. *Active Movers* classes.

**Conclusion:**

Activity patterns characterised by high sedentary behaviour were associated with specific pain characteristics in older adults, particularly among women and the oldest-old. Longitudinal studies are needed to clarify the direction of these associations.

**Supplementary Information:**

The online version contains supplementary material available at 10.1186/s11556-026-00422-0.

## Background

Physical activity (PA) is increasingly recognised as a key factor in health promotion and disease prevention, reducing the risk of chronic conditions and enhancing subjective health outcomes. These benefits have been observed across different intensities of PA, including moderate-to-vigorous physical activity and light physical activity [1–3]. In contrast, sedentary behaviour is associated with increased risk of morbidity and mortality [4]. Although individual movement behaviours are independently associated with health outcomes, their risks and benefits may also depend on how these behaviours are distributed and combined across waking hours. This suggests that examining integrated behavioural patterns may provide additional insight beyond analysing each behaviour separately [5, 6]. Consequently, examining combined patterns of PA and sedentary behaviour—referred to as activity patterns [7]—may provide a more comprehensive understanding on their influence on symptoms and health outcomes. In this context, person-centred methodological approaches such as latent class analysis (LCA) are well suited to capture heterogeneity in how individuals accumulate and combine movement behaviours across the day. This approach allows for the characterisation of real-world activity patterns—such as individuals who are both highly sedentary and highly physically active—that would not be captured when examining each behaviour in isolation [6].

Pain can be defined as “*an unpleasant sensory and emotional experience associated with*,* or resembling that associated with*,* actual or potential tissue damage”* [8]. It is a subjective experience shaped by biological, psychological, and social factors, and influenced by prior lived experiences [8]. However, pain constitutes an umbrella concept, within which distinct pain characteristics (i.e. intensity, duration and frequency of pain) are commonly evaluated to gain a clearer overview of the pain profile [9]. Also, pain represents a major public health concern, given its high prevalence and societal costs [10]. The prevalence of pain increases with age, affecting approximately 28% of the adult population [11] and between 36% and 71% of older adults [12, 13].

Previous research suggests that pain may influence the accumulation of PA and sedentary behaviour during the day. Experiencing pain during movement has been shown to promote more conservative movement strategies [14], and pain associated with specific movements may alter overall activity patterns and give rise to fear-avoidance behaviour [15]. These adaptations may have implications for habitual activity patterns, particularly in older adults as a result of their high pain prevalence.

Compared to younger age groups, older adults generally spend a larger proportion of their time in sedentary behaviour and accumulate less moderate-to-vigorous physical activity [16, 17]. Furthermore, overall, PA volumes tend to decline with advancing age, resulting in pronounced differences between younger-old and oldest-old adults [18]. Sex differences have also been reported, although findings remain inconsistent. While self-reported data often suggest that women engage in lower volumes of PA, evidence from device-measured assessments indicate higher overall PA volumes among women, largely due to more time spent in light physical activity [17, 19].

Sex differences have also been observed in pain characteristics in older adults. Compared with men, women generally report more severe pain and pain affecting multiple locations [12], and female sex has been described as a risk factor for musculoskeletal pain [20]. However, a previous study found that older men experiencing daily pain had an increased risk of injurious falls over 3-and 10 year follow-up periods, whereas no such association was observed in older women [21]. This finding indicates that movement adaptations in response to pain may be influenced by sex.

However, there is little evidence on how pain is related to device-measured activity patterns, rather than isolated PA or sedentary behaviour metrics [20]. Thus, there is a need for further research examining how pain relates to habitual activity patterns in older adults, with particular attention to potential differences by age and sex. Therefore, this study examined the associations between pain characteristics and device-measured activity patterns—integrating PA and sedentary behaviour metrics through LCA—in older adults and assessed whether and how these associations were modified by age and sex.

## Materials and methods

### Study sample

The present study used cross-sectional data from the Swedish National Study of Aging and Care-Kungsholmen (SNAC-K), a population-based cohort of older adults living in Kungsholmen, Stockholm, Sweden. The baseline examinations, conducted between 2001 and 2004, comprised 3363 participants (73% response rate) across eleven age groups: 60, 66, 72, 78, 81, 84, 87, 90, 93, 96, 99 + years [22]. At baseline, participants underwent comprehensive assessments, including face-to-face interviews, clinical examinations, and physical and cognitive testing performed by physicians, nurses and psychologists. Follow-up assessments are conducted every sixth year for younger cohorts (aged ≤ 78 years) and every third year for the older cohorts (aged ≥ 78 years) [17].

**Fig. 1:**
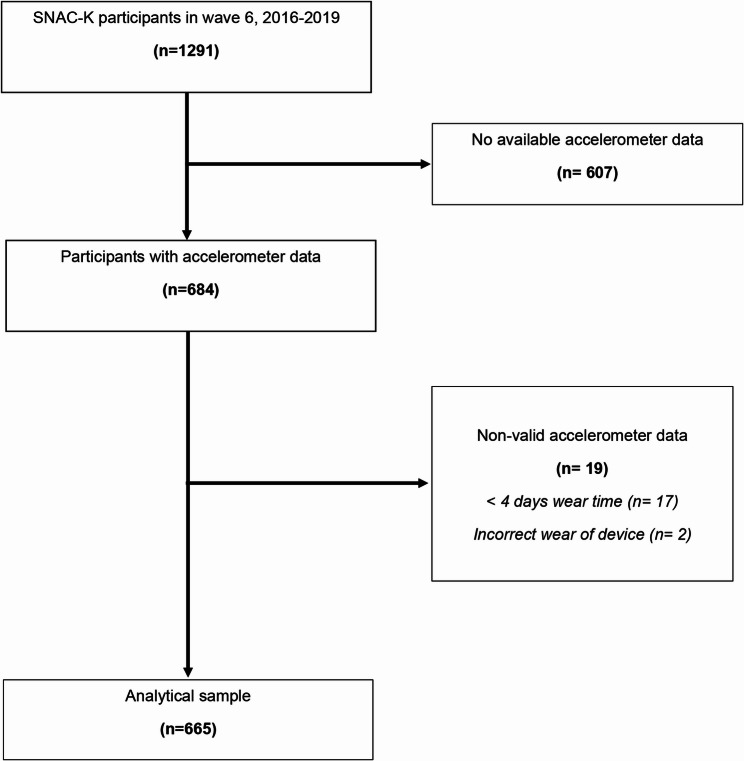
Flow-chart of included and excluded participants from SNAC-K wave 6, 2016–2019

The present study used data from the 6th wave of SNAC-K, (2016–2019) when collection of device-measured PA data was first introduced. Of the 1291 participants assessed in this wave, 684 met the eligibility criteria (no severe cognitive impairment, and able to move indoors without assistance) and consented to wearing an accelerometer for 7 consecutive days. In accordance with the predefined criteria and recommendations [23], nineteen participants were excluded due to having less than 4 days of valid wear time (*n* = 17) or improper device use (*n* = 2), leaving an analytical sample of 665 participants (Fig. 1). The SNAC-K study has received ethical approval from the Swedish Ethical Review Authority, and participants provided written informed consent. Compared to the analytical sample, individuals who did not participate in the accelerometer assessment (*n* = 607) were on average older, had more chronic conditions and lower levels of physical function (Supplementary Table S1).

### Data collection

#### Accelerometer assessment

PA and sedentary behaviour were assessed using the activPAL3™ accelerometer (PAL Technologies Ltd., Glasgow, UK). The accelerometer was placed on the participants’ thigh the day after their SNAC-K nurse assessment. They were instructed to wear it during all waking hours for seven consecutive days, excluding water-based activities like swimming or showering. Following the main recommendations [23], data were considered valid if the device was worn for at least 10 h per day on a minimum of four consecutive days. The activPAL3™ has been validated for measuring body posture and movement, including time spent sitting or standing, step count, step cadence (intensity of PA) and transitions from sitting to standing [24, 25] .

Data processing was performed using Excel macro-HSC PAL 2.21 analysis software, developed by Dr Philippa Dall and Professor Malcolm Granat, Faculty of Health and Life Sciences, Glasgow Caledonian University [26] (Supplementary Text S1). Step cadence, derived from the device’s imbedded algorithm, was used to determine PA intensity. A validated threshold of ≥ 100 steps per minute was applied to classify moderate-to-vigorous physical activity, while < 100 steps per minute (including standing) was defined as light physical activity [27]. Light- and moderate-to-vigorous physical activity events were defined as continuous walking episodes of at least two consecutive steps at the respective intensity.

The study included the following continuous variables: number of steps, time spent in light physical activity, number of light physical activity events, time spent in moderate-to-vigorous physical activity, number of moderate-to-vigorous physical activity events, time spent in sedentary behaviour, average duration of sedentary bouts. These variables were selected to capture habitual activity patterns, integrating overall time spent in both PA and sedentary behaviour, as well as accumulation metrics, such as frequency and bout duration for each behaviour [7].

#### Pain characteristics

Pain was assessed following the recommendations by the American Geriatric Society, emphasizing the importance of capturing pain location, intensity, and frequency [9]. Participants completed a questionnaire at the time of the physician’s assessment, reporting on their experiences of pain during the 4 weeks preceding the interview. The questionnaire included items on the presence of pain, pain frequency, number of pain locations and pain intensity [21]. The presence of pain was assessed with the question, “In the last 4 weeks, have you experienced pain?”. Response options were “yes” and “no.” Pain location was assessed using a 9-item question that asked whether the individual experienced pain in the head, neck, back, joints, shoulders/upper extremities, lower extremities/feet, chest, abdomen, or genitals. Pain intensity was measured with the question, “In the last 4 weeks, how much pain have you had?”. Available response options were “none,” “some,” “mild,” “moderate,” “moderate to severe,” “severe,” and “very severe.” Pain frequency was assessed by asking, “In the last 4 weeks, how often have you had pain?”. Response options included “once or twice,” “a few times,” “quite often,” “very often,” “daily,” or “almost daily.”

For the analysis, *presence of pain* was categorised as no or yes; *pain frequency* as almost never, sometimes and daily/almost daily; *pain intensity* as none, mild to moderate or moderately severe to very severe; *number of pain locations* as single or multiple (1 or ≥ 2 locations, bearing in mind that individuals reporting joint pain were classified as having pain at ≥ 2 locations). For the construction of the severity score, an additional total pain severity variable was calculated by summing the four pain characteristics [presence of; 0–1, intensity; 0–2, frequency; 0–2, locations; 0–2] ranging from 0 (no pain) to 7 (highest severity). The total pain severity score was further categorised as low/moderate severity (< 6) or high severity (≥ 6). This approach has been previously described in literature [28].

#### Activity patterns

Latent class analysis (LCA) was conducted to identify underlying activity patterns among the participants, based on the included PA and sedentary behaviour variables. LCA is a person-centred, data-driven method that uses mixture modelling to classify individuals into mutually exclusive latent classes based on multiple indicators, while accounting for measurement error and probabilistic class membership. This approach is well suited for identifying meaningful behavioural subgroups in large population-based samples [29]. Prior to the analysis, continuous PA variables were categorised to fit the LCA model: time spent in light physical activity, number of light physical activity events, number of moderate-to-vigorous physical activity events, and average duration of sedentary behaviour bouts were dichotomised at the median; time spent in moderate-to-vigorous physical activity and sedentary behaviour were divided into tertiles; number of steps was dichotomised using a threshold of 8000 steps per day, based on previous literature [30]. For a full description of the PA/sedentary behaviour variable categorisation, see Supplementary Table S2.

Among the 665 participants, three latent classes were identified: *Active Movers* (*n* = 282), *Balanced Movers* (*n* = 138) and *Sedentary Movers* (*n* = 245). Although, the Bayesian Information Criterion (BIC) suggested that a four-latent-class model provided the best model fit (Supplementary Table S3), inspection of the four latent classes (Supplementary Figure S1) revealed that two and three were similar, which could complicate clinical interpretation. Therefore, a three-class model was selected, providing interpretability and adequate sample sizes across classes for the subsequent regression analysis.

**Fig. 2:**
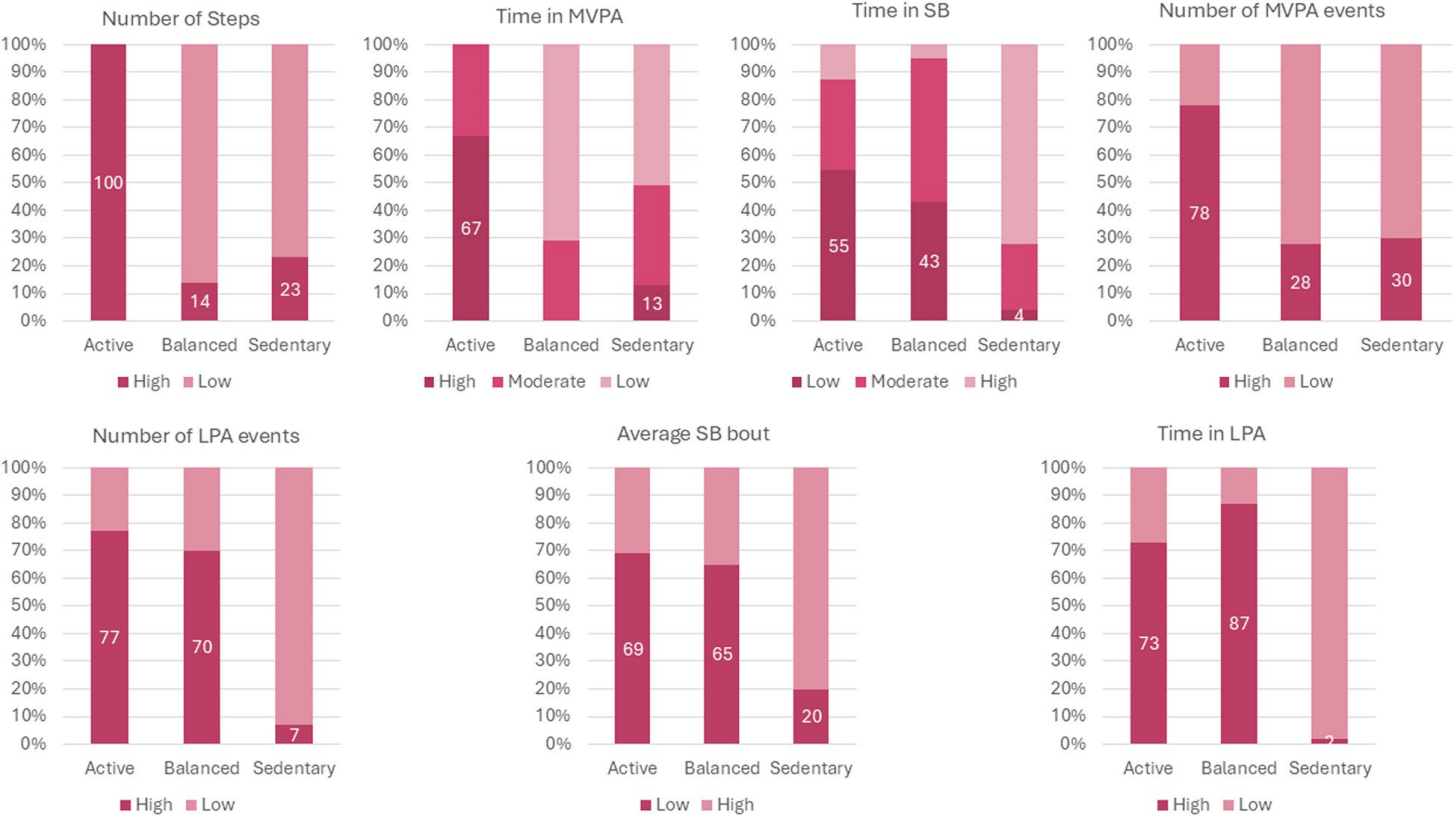
Distribution of activity variables across latent classes of activity pattern. MVPA= Moderate-to-vigorous physical activity, LPA= Light physical activity, SB = Sedentary behaviour

*Active Movers* exhibited high overall PA, with substantial time spent in both moderate-to-vigorous physical activity and light physical activity. *Balanced Movers* showed moderate overall PA, with high duration of light physical activity but low duration of moderate-to-vigorous physical activity, and the lowest frequency of moderate-to-vigorous physical activity events across classes. *Sedentary Movers* were the least active, spending the least time in light physical activity and the most time in sedentary behaviour, both in total time and bout duration across classes (Fig. 2). Additional analyses that address sex and age differences within the activity classes are displayed in the Supplementary material (Supplementary Figure S2 and S3).

### Covariates

The analysis included the following covariates: sex (woman, man), age (date of birth), educational level (elementary, high school, university), body mass index (BMI; <20, 20-24.9, 25-29.9, ≥ 30 kg/m^2^), co-habitation status (living alone, co-habiting), physical function, assessed as the ability to perform five consecutive chair stands (yes/ no), number of chronic diseases and depressive symptoms, measured using the Montgomery-Åsberg Depression Scale (< 9/ ≥ 9). The method for operationalizing the number of chronic diseases has been described elsewhere [31], and the cut-off for depressive symptoms has been previously used in research on older adults [32].

### Statistical analysis

Multinomial logistic regression models were used to assess the association between the latent activity classes identified by the LCA model (*Active Movers*,* Balanced Movers* and *Sedentary Movers*) as the dependent variables and each pain characteristic (presence of pain; pain frequency; pain intensity; number of pain locations and the summary measure of pain severity) as independent variables. The activity class with the highest PA volume (*Active Movers*) served as the reference category. A two-model approach was performed to adjust for potential confounders. Model 1, adjusted for age, sex and educational level, while Model 2 further adjusted for co-habitation status, physical function, number of chronic diseases, BMI and depressive symptoms.

To explore potential age and sex differences, multiplicative interactions were tested by incorporating the product of age category (= 66 years/ ≥80 years) or biological sex (male/female) and each pain characteristic into the models.

Several sensitivity analyses were performed. First, multinomial regression analyses were repeated after categorising participants into four latent classes. Second, analyses were repeated using *Balanced Movers* (instead of *Active Movers*) as the reference group. Third, the potential modifying effect of having at least one musculoskeletal disease was examined by including the interaction terms between musculoskeletal disease [categorised based on previous operationalization [31] as inflammatory arthropathies, osteoarthritis and other degenerative joint diseases, dorsopathies, osteoporosis, other musculoskeletal and joint disorders] (yes/ no) and each pain characteristic in the regression model. Fourth, the same approach was used to evaluate the potential modifying effect of analgesic drug [categorised based on the Anatomical Therapeutic Chemical (ATC) classification system codes N02 (analgesic) and M01A (NSAID) excluding glucosamine M01AX05] use (yes/no). Missing data on independent variables was assessed to be highly limited and were not expected to affect the analyses substantially.

The study analysis plan was uploaded on the Open Science Framework platform (osf.io) prior to, and during the analysis process to ensure transparency throughout study process (Lager C. Exploring movement behaviour patterns and pain associations in older adults: the role of age and sex. OSF; 2026 Jan 29. Available from: https://osf.io/pujdk).

All statistical analyses were performed with STATA 18.0 (Stata-Corp LP, College Station, TX, USA).

## Results

### Characteristics of the study population

The median age of the total sample was 66.5 (IQR: 66.1, 81.2), and a majority of participants were female (64%), co-habiting (55%), and had a university degree (58%). Across the latent classes, the *Active Movers* had the highest proportion of younger-old (median age 66.1, IQR: 66.0, 67.1), while *Balanced Movers* had the highest proportion of oldest-old (median age 81.0, IQR: 66.3, 84.1). The average number of daily steps were 8680 ± 3753 in the total sample, 11,834 ± 2821 among *Active Movers*, 6597 ± 1830 among *Balanced Movers* and 6223 ± 2696 among *Sedentary Movers* (Table 1). *Sedentary Movers* had an overall higher prevalence across all pain characteristics than the other classes. However, pain severity was comparable between *Balanced* and *Sedentary Movers* (Supplementary Table S4).

**Table 1:** Characteristics of participants in the total sample and by latent class

Characteristics	Total (*n* = 665)	Active Movers (*n* = 282)		Balanced Movers (*n* = 138)	Sedentary Movers (*n* = 245)
Proportion of younger-old (= 66 years), %	57	78		36	46
Age, Median (IQR)	66.5 (66.1, 81.2)	66.1 (66.0, 67.1)		81.0 (66.3, 84.1)	80.1 (66.1, 84.1)
Women, n (%)	426 (64)	189 (67)		96 (70)	141 (58)
Educational level, n (%)					
Elementary or High School	281 (42)	92 (33)		76 (55)	113 (46)
University	384 (58)	190 (67)		62 (45)	132 (54)
Co habitation status, n (%)					
Living alone	296 (45)	104 (37)		62 (45)	130 (53)
Married/co- living, n (%)	369 (55)	178 (63)		76 (55)	115 (47)
Body Mass Index, Mean (SD)	25.6 (3.9)	24.8 (3.4)		26.3 (4.3)	26.3 (4.2)
Chair stand test, n able to do 5 chair stands (%)	597 (90)	274 (98)		119 (88)	204 (84)
Depressive symptoms, YES, n (%)	29 (4.3)	11 (3.9)		7 (5.1)	11 (4.5)
Use of analgesic drugs, YES, n (%)	229 (34.4)	82 (29.1)		49 (35.5)	98 (40.0)
Number of chronic diseases, Mean (SD)	4.9 (2.9)	3.7 (2.2)		5.9 (3.1)	5.7 (3.0)
Number of steps/day, Mean (SD)	8680 (3753)	11,834 (2821)		6597 (1830)	6223 (2696)
Daily time spent in Sedentary behaviour (hours), Mean (SD)	8.8 (1.6)	7.9 (1.4)	8.2 (1.0)		10.1 (1.2)
Average Sedentary behaviour-bout duration (minutes), Mean (SD)	13.4 (8.6)	10.8 (3.0)		11.8 (3.8)	17.2 (12.6)
Daily time spent in Light physical activity (minutes), Mean (SD)	305.0 (95.2)	348.7 (92.3)		355.3 (59.4)	226.3 (54.8)
Daily time spent in Moderate-to-vigorous physical activity (minutes), Mean (SD)	31.5 (25.8)	50.4 (23.6)		12.2 (9.3)	20.6 (19.3)
Light physical activity walking events (n), Mean (SD)	322.5 (103.3)	380.3 (95.9)		351.5 (77.0)	239.7 (62.4)
Moderate-to-vigorous physical activity walking event (n), Mean (SD)	41.4 (27.0)	57.9 (25.8)		29.2 (19.9)	29.3(21.3)

Missing: *1 Body Mass Index, 2 smoking status, 8 number of chronic diseases, 5 chair-stand test

### Associations between pain characteristics and activity patterns

In the fully adjusted multinomial regression model, moderately severe to very severe pain was associated with membership in the *Sedentary vs. Active Movers* class in the total sample (RRR: 2.16, 95% CI: 1.06, 4.40, *p* = 0.034). No evident associations were observed between pain characteristics and the *Balanced vs. Active Movers* class in either model (Table 2).

**Table 2:** Relative Risk Ratio (RRR) and 95% Confidence Intervals (CI) for pain characteristics across three latent classes of activity pattern, total sample

Relative Risk Ratio (95% CI) from multinominal regression analysis										
Pain characteristics	Model 1					Model 2				
	**Active Movers** (reference)	**Balanced Movers** (*n* = 138)	***p*** **-value**	**Sedentary Movers** (*n* = 245)	***p*** **-value**	**Active Movers** (reference)	**Balanced Movers** (*n* = 138)	***p*** **-value**	**Sedentary Movers** (*n* = 245)	***p*** **-value**
Pain prevalence										
* Yes*	1.0	1.36 (0.85, 2.16)	0.201	1.82 (1.23, 2.69)	*0.003*	1.0	1.04 (0.63, 1.72)	0.874	1.42 (0.92, 2.18)	0.113
Pain intensity	1.0					1.0				
* Mild to Moderate*	1.0	1.30 (0.78, 2.18)	0.313	1.46 (0.94, 2.27)	0.090	1.0	1.08 (0.62, 1.87)	0.790	1.24 (0.77, 2.00)	0.385
* Moderately severe to Very severe*	1.0	1.71 (0.77, 3.79)	0.188	3.44 (1.78, 6.63)	*< 0.001*	1.0	1.11 (0.48, 2.57)	0.816	2.16 (1.06, 4.40)	*0.034*
Pain Frequency										
* Sometimes*	1.0	1.37 (0.74, 2.56)	0.321	1.36 (0.79, 2.34)	0.263	1.0	1.13 (0.59, 2.17)	0.709	1.24 (0.70, 2.20)	0.470
* Daily/almost daily*	1.0	1.46 (0.81, 2.62)	0.204	2.31 (1.42, 3.76)	*0.001*	1.0	0.99 (0.53, 1.87)	0.978	1.53 (0.89, 2.61)	0.123
Pain Locations										
* Single*	1.0	1.51 (0.84, 2.74)	0.171	1.92 (1.17, 3.15)	0.009	1.0	1.21 (0.65, 2.25)	0.545	1.61 (0.95, 2.72)	0.079
* Multiple*	1.0	1.32 (0.71, 2.42)	0.378	1.77 (1.05, 2.97)	*0.031*	1.0	0.91 (0.47, 1.75)	0.768	1.23 (0.69, 2.19)	0.476
Pain Severity *High*	1.0	1.75 (1.06, 2.89)	*0.029*	2.12 (1.38, 3.26)	*0.001*	1.0	1.26 (0.74, 2.16)	0.400	1.51 (0.94, 2.41)	0.089

Model 1 adjusted for sex, age and educational level. Model 2 additionally adjusted for co-habitation status, BMI, physical function, number of chronic diseases and depressive symptoms

Results from the fully adjusted interaction analysis by age category (= 66 years/ ≥80 years) showed an association between pain prevalence (RRR: 2.27, 95% CI: 1.10, 4.68, *p* = 0.027) daily pain (RRR: 3.43, CI: 1.28, 9.24, *p* = 0.015) and pain in a single location (RRR: 3.24, 95% CI: 1.15, 9.08, *p* = 0.025) and membership in the *Sedentary Movers* class, among the oldest-old. Consistent with previous analyses, no evident relationship was found between pain characteristics and the *Balanced vs. Active Movers* class (Table 3).

**Table 3:** Relative Risk Ratio (RRR) and 95% Confidence Intervals (CI) for pain characteristics across latent classes of activity pattern, by age group

Pain characteristics	Relative Risk Ratio (95% CI) from multinominal regression analysis								
			**Younger-old** (*n* = 382) *Active Movers* (*n* = 219)				**Oldest- old** (*n* = 283) *Active Movers* (*n* = 63)		
	**Active Movers** (reference)	**Balanced Movers** (*n* = 50)	***p*** **-value**	**Sedentary Movers** (*n* = 113)	***p*** **-value**	**Balanced Movers** (*n* = 88)	***p*** **-value**	**Sedentary Movers** (*n* = 132)	***p*** **-value**
Pain previous 4 weeks									
* Yes*	1.0	1.00 (0.51, 1.98)	0.998	0.89 (0.52, 1.52)	0.675	1.41 (0.65, 3.05)	0.389	2.27 (1.10, 4.68)	*0.027*
Pain intensity									
* Mild to Moderate*	1.0	0.79 (0.36, 1.76)	0.571	0.85 (0.47, 1.55)	0.598	1.77 (0.75, 4.17)	0.192	2.04 (0.90, 4.62)	0.086
* Moderately severe to Very severe*		1.77 (0.63, 4.99)	0.278	1.19 (0.49, 2.91)	0.699	0.81 (0.21, 3.19)	0.764	2.56 (0.77, 8.53)	0.126
Pain frequency									
* Sometimes*	1.0	1.17 (0.48, 2.84)	0.723	1.02 (0.50, 2.07)	0.965	1.16 (0.43, 3.13)	0.766	1.42 (0.56, 3.65)	0.461
* Daily/almost daily*	1.0	0.90 (0.38, 2.15)	0.819	0.78 (0.39, 1.54)	0.470	1.85 (0.64, 5.34)	0.255	3.43 (1.28, 9.24)	*0.015*
Pain locations									
* Single*	1.0	1.22 (0.55, 2.70)	0.619	1.01 (0.53, 1.91)	0.978	1.71 (0.56, 5.22)	0.343	3.24 (1.15, 9.08)	*0.025*
* Multiple*	1.0	0.75 (0.27, 2.07)	0.582	0.78 (0.36, 1.66)	0.513	1.22 (0.48, 3.09)	0.675	1.70 (0.71, 4.06)	0.235
Pain Severity *High*	1.0	1.40 (0.67, 2.90)	0.371	1.21 (0.66, 2.22)	0.528	1.38 (0.60, 3.13)	0.447	2.11 (0.97, 4.56)	0.058

All models adjusted for sex, age, educational level, co-habitation status, BMI, physical function, number of chronic diseases and depressive symptoms

### Interaction analyses with sex and age

The interaction analysis with sex showed that, in the fully adjusted model, women reporting pain prevalence (RRR: 1.75, 95% CI: 1.04, 2.96, *p* = 0.036), moderately severe to very severe pain (RRR: 3.07, 95% CI: 1.38, 6.84, *p* = 0.006), daily pain (RRR: 2.12, 95% CI: 1.13, 3.98, *p* = 0.019), or high pain severity (RRR: 2.06, 95% CI: 1.17, 3.62, *p* = 0.012) were more likely classified as *Sedentary vs. Active Movers* than men. No evident relationship was found between pain characteristics and belonging to the *Balanced vs. Active Movers* class in the sex interaction analysis (Table 4).

**Table 4:** Relative Risk Ratio (RRR) and 95% Confidence Intervals (CI) for pain characteristics across latent classes of activity pattern, by sex

Relative Risk Ratio (95% CI) from multinominal regression analysis									
		**Men** (*n* = 239) *Active Movers* (*n* = 93)				**Women** (*n* = 426) Active Movers (*n* = 189)			
Pain characteristics	**Active Movers** (reference)	**Balanced Movers** (*n* = 42)	***p*** **-value**	**Sedentary Movers** (*n* = 104)	***p*** **-value**	**Balanced Movers** (*n* = 96)	***p*** **-value**	**Sedentary Movers** (*n* = 141)	***p*** **-value**
Pain previous 4 weeks									
Yes	1.0	1.02 (0.41, 2.52)	0.967	0.94 (0.45, 1.96)	0.871	1.07 (0.60, 1.92)	0.823	1.75 (1.04, 2.96)	*0.036*
Pain intensity									
Mild to Moderate	1.0	1.14 (0.43, 3.06)	0.790	1.05 (0.48, 2.32)	0.901	1.07 (0.56, 2.05)	0.847	1.39 (0.76, 2.52)	0.281
Moderately severe to Very severe		0.62 (0.11, 3.50)	0.585	0.59 (0.13, 2.57)	0.478	1.27 (0.49, 3.28)	0.626	3.07 (1.38, 6.84)	*0.006*
Pain frequency									
Sometimes	1.0	1.44 (0.46, 4.54)	0.533	1.35 (0.52, 3.53)	0.541	1.03 (0.47, 2.25)	0.948	1.22 (0.59, 2.51)	0.594
Daily/almost daily	1.0	0.68 (0.20, 2.37)	0.547	0.63 (0.24, 1.69)	0.360	1.15 (0.56, 2.37)	0.699	2.12 (1.13, 3.98)	*0.019*
Pain locations									
Single	1.0	1.38 (0.47, 4.09)	0.559	1.39 (0.57, 3.38)	0.468	1.15 (0.55, 2.45)	0.707	1.81 (0.93, 3.49)	0.078
Multiple	1.0	0.62 (0.16, 2.40)	0.490	0.48 (0.16, 1.46)	0.197	1.04 (0.50, 2.20)	0.908	1.73 (0.89, 3.36)	0.108
Pain Severity									
*High*	1.0	0.90 (0.33, 2.44)	0.834	0.75 (0.33, 1.70)	0.489	1.46 (0.78, 2.74)	0.233	2.06 (1.17, 3.62)	*0.012*

All models adjusted for sex, age, educational level, co-habitation status, BMI, physical function, number of chronic diseases and depressive symptoms.

The multinomial regression models including the product of sex and pain characteristics showed a significant interaction for moderately severe to very severe pain intensity (*p* = 0.032) and suggestive evidence for daily pain (*p* = 0.070) among the *Sedentary Movers* class while models including the product of age category (= 66 years/ ≥80 years) revealed a significant interaction for pain prevalence (*p* = 0.036) and daily pain (*p* = 0.009). No significant interactions were found between age or sex and pain characteristics among the *Balanced Movers* class (Supplementary Table S5).

### Sensitivity analysis

In the first sensitivity analysis, exploring the association between pain characteristics and four latent PA classes, the LCA model primarily differentiates the *Balanced Movers* into two groups that share similar attributes, yet one group demonstrates greater amounts of moderate-to-vigorous physical activity, and a higher step count compared to the other (Supplementary Figure S2). The multinomial regressions displayed similar results as the main analysis, although no evident relationship was observed in the fully adjusted model (Supplementary Table S6). Interaction analysis with the product of age group or sex and pain also produced similar results, with evident interactions observed for membership in the most sedentary class (Supplementary Table S7 and S8).

In the second sensitivity analysis, replacing *Active Movers* with *Balanced Movers* as the reference category did not reveal any significant associations in the total sample (Supplementary Table S9). The sex-interaction analysis presented only a single association of higher pain intensity and membership in the *Sedentary vs. Balanced Movers* class (RRR: 2.42, 95% CI: 1.09, 5.37) for women. In the age-interaction analysis, the results suggested an association of higher pain intensity (RRR: 3.16, 95% CI: 1.24, 8.06) and probability of membership in the *Sedentary vs. Balanced Movers* class only in the oldest-old. No evident relationship was found between pain characteristics and the *Active Movers* class (Supplementary Table S10). The sensitivity analysis examining the potentially modifying effect of having at least one musculoskeletal disease indicated that individuals with a musculoskeletal disorder and moderately severe to very severe pain, were more likely to belong to the *Sedentary vs. Active Movers* class (RRR: 2.36, 95% CI: 1.02, 5.49). No evidence of association was found between the use of analgesic drugs on pain reporting and class membership (Supplementary Table S11).

## Discussion

In this cohort of generally well-functioning older adults, three distinct activity classes were identified: *Active Movers*,* Balanced Movers*,* and Sedentary Movers*. Certain pain characteristics were linked to membership in the *Sedentary Movers vs. Active Movers* class, particularly among the oldest-old and in women.

*Active Movers* showed an overall high volume of PA, with a high duration of moderate-to-vigorous physical activity per day. This class also showed the lowest proportion of pain experience across all activity classes. Meanwhile, *Balanced Movers* and *Sedentary Movers* had similar volumes of PA and number of moderate-to-vigorous physical activity events. Notably, although *Sedentary Movers* appear to engage in slightly more time in moderate-to-vigorous physical activity compared to *Balanced Movers*, they allocate only a minimal amount of time to light physical activity—and do so less often—relative to the other classes. This could be an indication of a limited engagement in standing activities and performance of everyday household tasks— activities that typically occur within light physical activity. In addition, the *Sedentary Movers* spent approximately 1.9 h more in sedentary behaviour per day compared to the *Balanced Movers*, indicating a more prolonged behaviour of sedentary activities.

In the total sample, higher pain intensity was associated with membership in the *Sedentary Movers* class. This aligns with findings from a Chinese study exploring the relationship between pain characteristics and self-reported activity patterns, which reported that higher pain intensity was associated with activity patterns of avoidance and pacing, particularly among older adults with chronic musculoskeletal pain [33]. Other studies have similarly reported lower PA levels among older adults with chronic pain [34]. Although, our study did not distinguish between chronic and non-chronic pain, our sensitivity analysis suggests that having a musculoskeletal disorder—likely reflecting predominantly chronic- or long term conditions based on our operationalisation (e.g., osteoarthritis, inflammatory arthropathies, and degenerative spinal conditions)—combined with higher pain intensity may be associated with a more sedentary pattern. In addition, kinesiophobia (fear of movement) has been identified as a factor associated with physical impairment and lower levels of PA, particularly among older adults [35]. Although sedentary behaviour may be an unavoidable and even desirable effect of acute pain, prolonged sedentary behaviour is associated with detrimental health outcomes [36]. Hence, how to cope with- and adapt activity patterns to the personal experience of pain is an important task for health care providers— and should potentially focus on how to limit the amount of sedentary behaviour.

No association between pain characteristics and activity pattern was evident among *Balanced Movers*, although participants with this activity pattern spent less overall time in moderate-to-vigorous physical activity than *Sedentary Movers.* In contrast, a previous study reported that chronic widespread pain was associated with spending less time in moderate-to-vigorous physical activity, but no differences in sedentary time. These conflicting results are likely due to methodological differences in assessing PA, sedentary behaviour, and the fact that our study did not distinguish chronic widespread pain and other forms of pain.

The relationship between pain and activity patterns is likely bidirectional, as PA has been shown to influence pain modulation. For example, higher overall PA may enhance pain inhibition [37], and different intensities of PA appear to affect pain differently, with moderate-to-vigorous physical activity being particularly effective in reducing experimentally measured pain, such as pressure pain thresholds [38]. Therefore, while our findings suggest that pain characteristics may influence activity patterns, it is also possible that specific activity patterns, in turn, modulate pain. For example, greater amounts of sedentary behaviour are associated with a higher risk of neck pain, with risk increasing alongside duration [39]. In addition, we found no association between pain and activity patterns depending on whether analgesic drugs were used or not. However, variations in the type of medication taken, dosage, regularity of use, and individual responses to analgesic treatment are likely to play an important role [40]. Consequently, underreporting of pain may occur if individuals regularly use analgesics that result in absence of pain.

Following the age interaction analyses, we found evidence suggesting an association that, particularly among the oldest-old, the presence of pain, daily pain, and pain in a single location were associated with belonging to the *Sedentary vs. Active Movers* class. Similar to the sex-specific interaction analyses, a relationship between high-intensity pain and the *Sedentary Movers* class in the oldest-old emerged when the reference category was changed to *Balanced Movers*. These findings are consistent with a previous study by Zhou et al., which examined the relationship between sedentary patterns and health outcomes in adults aged 80 years and older. In that study, higher volumes of sedentary behaviour were associated with greater number of difficulties, including pain, during the performance of daily activities [41]. Moreover, participants with higher sedentary time had, on average, higher anxiety levels and more limitations in activities of daily living compared to those who were less sedentary [41]. Following this statement, lower levels of individual and social well-being have been seen to be associated with high volumes of sedentary behaviour for the oldest- old [42]. Given the evidence of detrimental health outcomes following prolonged sedentary behaviour [43], identifying strategies to remain physically active despite pain is an important health message, especially for the oldest-old since the prevalence of pain has been reported as high as 71% in this age-group [13].

In the sex-specific interaction analysis, we found that women reporting pain—whether in prevalence, higher intensity, or daily experience—were more likely to belong to the *Sedentary vs. Active Movers* class than men. This suggests that women with pain symptoms tend to adapt a more sedentary pattern. Notably, the association remained significant even when the reference category was changed to *Balanced Movers* in a sensitivity analysis, indicating that pain intensity is more closely linked to time spent in sedentary behaviour in women, regardless of overall PA volume. These results aligns with evidence that older women generally report higher prevalence and intensity of pain than men [12].

Several factors may contribute to these sex differences. Women generally exhibit higher pain sensitivity, including lower pain thresholds and tolerance [44], may be more prone to experience kinesiophobia in response to pain [45], and often experience greater limitations in physical function following pain, which can reduce mobility and daily activities [46]—potentially exacerbated by prolonged bouts of sedentary behaviour [47]. Although prolonged sedentary behaviour is linked to adverse health outcomes [48], increased sedentary time during pain may also serve a protective role, helping prevent symptom exacerbation or acute events [49]. For example, a previous study using data from the SNAC-K cohort found that older men who reported pain had an increased risk of injurious falls, whereas no such association was found in women [21].

In addition, older women and men appear to cope with pain differently. In a study by Serra et al. common coping strategies and pain intensity was assessed in 276 older men and women using the Brief Pain Inventory and Chronic Pain Coping Inventory. The authors reported that men more frequently used exercise as a coping strategy when experiencing intense pain, whereas this strategy was less commonly used by women [50]. In our study, associations between pain characteristics and the *Sedentary vs. Active Movers* activity pattern were evident in women but not in men, which may indicate sex differences in pain coping strategies. This could suggest that women may be more likely to modify their activity pattern in response to pain, potentially as an adaptive strategy to reduce the risk of injurious falls [21]. Nevertheless, both total time in sedentary behaviour and prolonged bouts of sedentary behaviour have been associated with adverse health outcomes, including decreased lower extremity muscle mass [51], and higher risks of morbidity and mortality [52]. Therefore, potential sex differences in activity pattern adaptations following pain should be addressed in future research, particularly through longitudinal studies examining differential risks for health outcomes. Moreover, given the established association between sedentary behaviour and adverse health effects, it is important to prevent increases in sedentary behaviour from becoming prolonged.

### Strengths and limitations

A major strength of the study is the use of data from the SNAC-K cohort, which enabled the inclusion of a large sample of older adults who underwent comprehensive and standardised health assessments by health professionals. Another notable strength is the use of device-measured PA through the activPAL3™ device, which minimizes the risk of misclassification and recall bias commonly associated with self-reported PA [53]. The activPAL3™ is also considered the gold standard device for the assessment of sedentary behaviour due to its ability to distinguish body posture and provide accurate positional data [25]. Another strength is the detailed categorisation of walking episodes, allowing both frequency- and intensity- related metrics to be incorporated in the analysis. Additionally, the use of 1-second epochs increases the precision of step count and cadence estimations, thereby enhancing the reliability of the derived activity pattern classifications [54].

Despite these strengths, several limitations should be acknowledged. Similar to other accelerometers, the activPAL3™ cannot capture non-ambulatory activities such as cycling or swimming. Moreover, the study participants were given instructions to wear the accelerometer during waking hours rather than wearing the accelerometer during the whole 24-hour cycle, which may have resulted in missing PA data, particularly during early morning and evening hours. Another important limitation is the cross-sectional design that precludes conclusions about the direction of relationship between pain characteristics and activity patterns. In addition, pain was assessed based on self-reports referring to the preceding 4 weeks, meaning that earlier pain experiences were not captured. Additionally, since SNAC-K was designed to include participants from specific predefined age cohorts (age 66, 81, 84, 87, 90, 93, 96 and 99 + years in the follow-up wave upon which the current study is based), we were limited in our ability to examine the effect of age as a continuous variable. Furthermore, participants included in the accelerometer assessment exhibited more favourable health profiles across most measured covariates compared with other SNAC-K participants; consequently, the observed associations may be underestimated, as individuals with more compromised health were underrepresented. This, together with the fact that the study population was drawn from a socioeconomically advantaged area, may limit the generalisability of the findings to the broader older population.

## Conclusion

In conclusion, specific pain characteristics were associated with a more sedentary activity pattern in older adults, particularly among women and the oldest age group. However, due to the cross-sectional study design, no conclusions can be drawn regarding the directionality of these associations. The findings suggest that women and individuals aged 80 years and older may require additional support to maintain an active lifestyle despite pain, in order to reduce the health risks associated with sedentary behaviour. At the same time, it is important to acknowledge that sedentary behaviour may, in some cases, represent an adaptive response to pain aimed at preventing further harm. To clarify causal pathways and directionality, future research should investigate the longitudinal relationship between pain and activity patterns.

## Supplementary Information

Supplementary Material 1.

## Data Availability

No datasets were generated or analysed during the current study.

## Abbreviations

LCA: Latent Class Analysis
PA: Physical Activity
SNAC-K: Swedish National study on Aging and Care- Kungsholmen
