# Supplementary material for: Pain and its association with device-measured activity patterns in older adults: age and sex differences in a population-based cross-sectional study
Source: Eur Rev Aging Phys Act. 2026 Jun 16;23:22. doi: 10.1186/s11556-026-00422-0 (PMC13289551; doi:10.1186/s11556-026-00422-0)
Supplement: Supplementary file 1 — Supplementary Material 1. [file 11556_2026_422_MOESM1_ESM.docx]

**Supplementary Table S1.** Comparison between included and non-included participants.

| Variable | Included  (n=665) | Non-included  (n=607) | P-value |
| --- | --- | --- | --- |
| Age (years), mean (SD) | 73.7 (9.0) | 82.1 (10.0) | *<0.001* |
| Women, n (%) | 426 (64) | 392 (65) | *0.725* |
| University-level education, n (%) | 384 (58) | 284 (47) | *<0.001* |
| Co-habitants, n (%) | 369(55.5) | 222 (38.2) | *<0.001* |
| Body mass index, mean (SD) | 25.6 (3.9) | 25.1 (4.4) | *0.014* |
| Depressive symptoms, n (%) | 29 (4) | 81 (13) | *<0.001* |
| Use of analgesic drugs, n (%) | 229 (34) | 285 (47) | *<0.001* |
| Able to perform chair stand test, n (%) | 597 (90) | 296 (51) | *<0.001* |
| Number of chronic diseases, mean (SD) | 4.9(2.9) | 11.7 (5.5) | *<0.001* |

Missing data; * 4 sex, 5 educational level, 26 co-habitation status, 32 chair stand test, 2 analgesic drugs, 2 depressive symptoms, 2 number of chronic diseases

**Supplementary Text S1.**

**Description of data extraction and data processing**

The activPAL3^TM^ records thigh position data to establish periods of sitting/lying, standing, walking, number of steps, and postural transitions with a sampling frequency of 20 Hz (1). To process the accelerometer data, the Excel macro-HSC PAL 2.21 analysis software, developed by Dr Philippa Dall and Professor Malcolm Granat, Faculty of Health and Life Sciences, Glasgow Caledonian University (2) was used. We used the CREA algorithm to extract data regarding the non-wear-time to set the HSC software for the data extraction with the VANE algorithm from the activPAL3^TM^ software manufacturer (PAL Analysis version 8, PAL Technologies Ltd, Glasgow, Scotland). Data extraction was conducted using 24-hour blocks (midnight to midnight), excluding half days, based on previous literature recommendations.

1. Edwardson CL, Winkler EAH, Bodicoat DH, Yates T, Davies MJ, Dunstan DW, et al. Considerations when using the activPAL monitor in field-based research with adult populations. J Sport Health Sci. 2017;6(2):162–78.

2. Iveson A, Granat M, Ellis B, Dall P. Concurrent Measurement of Global Positioning System and Event-Based Physical Activity Data: A Methodological Framework for Integration. Journal for the Measurement of Physical Behaviour. 2020;4:1–14.

The HSC software classified posture (based on inclination) and determined the duration and number of steps using 1 second epoch. The software combined individual walking steps (second-by-second) into single events and classified the intensity of these events based on cadence.

**Supplementary Table S2.** Description of included variables of movement behavior pattern

| Variable | Definition | Categorization |
| --- | --- | --- |
| Light physical activity (LPA) | Average time (minutes) of daily steps <100 steps per minute including standing | High= ≥301.5  Low= <301.5 |
| Moderate- to-vigorous physical activity (MVPA) | Average time (minutes) of daily steps ≥100 steps per minute | High= >37  Medium= ≥16.4, ≤ 37.0  Low= <16.4 |
| LPA Event | Walking event with a minimum of 2 steps, with a cadence <100 steps per minute, number | High= ≥320  Low= <320 |
| MVPA Event | Walking event with a minimum of 2 steps, with a cadence ≥100 steps per minute, number | High= ≥37.4  Low= <37.4 |
| Sedentary behavior (SB) | Average time spent in seated/reclining or lying position in hours | Low= < 8.2  Medium= ≥8.2, ≤9.4  High= >9.4 |
| Average sedentary bout | Average time of bout spent in seated/reclining or lying position in hours | Low= <12.0  High = 12.0 |
| Steps | Average number of total steps per day | High= ≥8000  Low= <8000 |

**Supplementary Table S3**. Bayesian information criterion and mean posterior probabilities of latent class analysis models.

| **Models** | **Bayesian information criterion** | **One latent class** | **Two latent classes** | **Three latent classes** | **Four latent classes** | **Five latent classes** |
| --- | --- | --- | --- | --- | --- | --- |
| One- latent class model | 7586.6 | 1.0 |  |  |  |  |
| Two-latent-class model | 6846.3 | 0.94 | 0.93 |  |  |  |
| Three-latent-class model | 6622.6 | 0.92 | 0.94 | 0.93 |  |  |
| Four-latent-class model | 6464.4 | 0.92 | 0.93 | 0.92 | 0.92 |  |
| Five-latent-class model | 6500.4 | 0.80 | 0.75 | 0.93 | 0.94 | 0.92 |

**
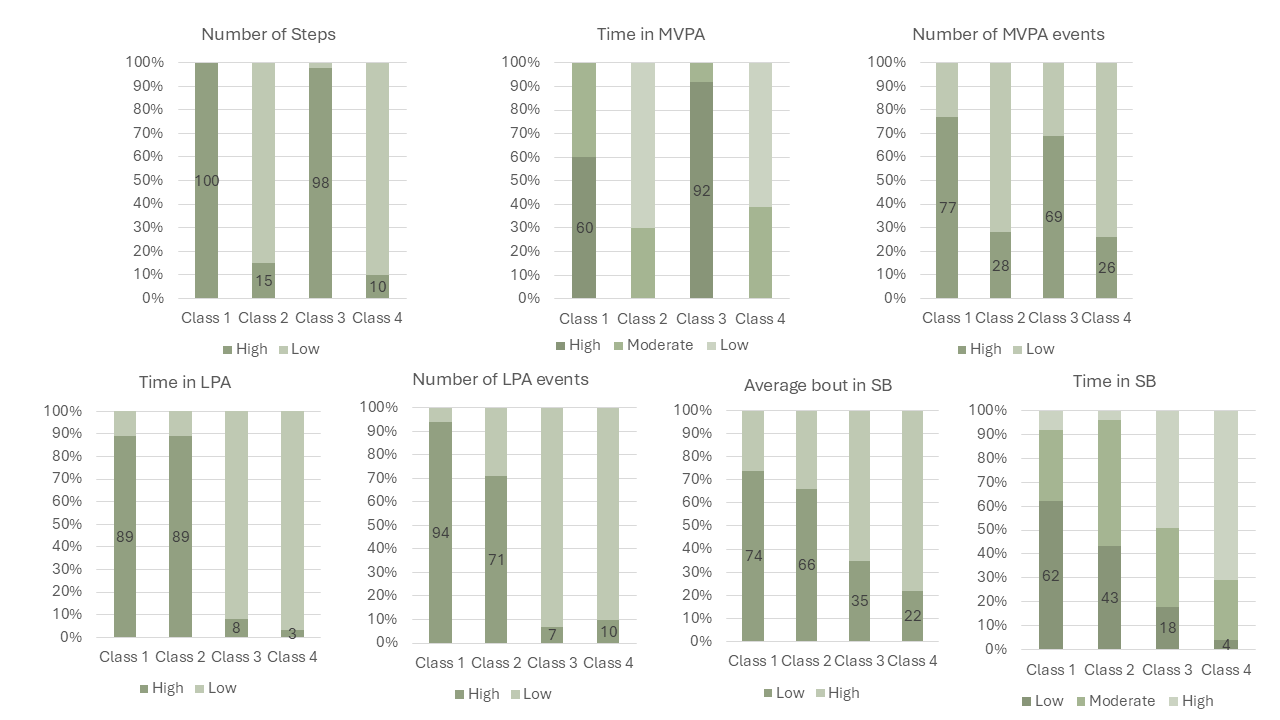
Supplementary Figure S1.** Distribution of physical activity variables for each of the four latent classes of activity pattern

**Supplementary Figure S2.** Distribution of activity variables by latent class and age group


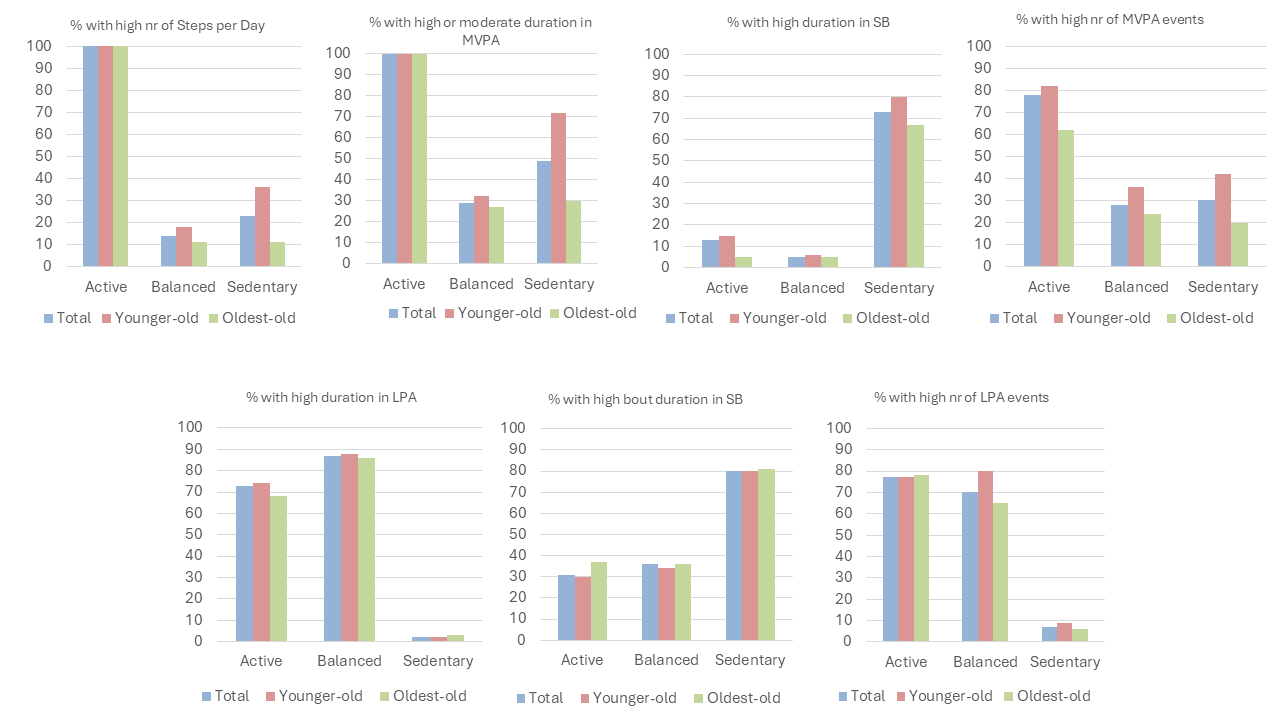


**Supplementary Figure S3.** Distribution of activity variables by latent class and sex


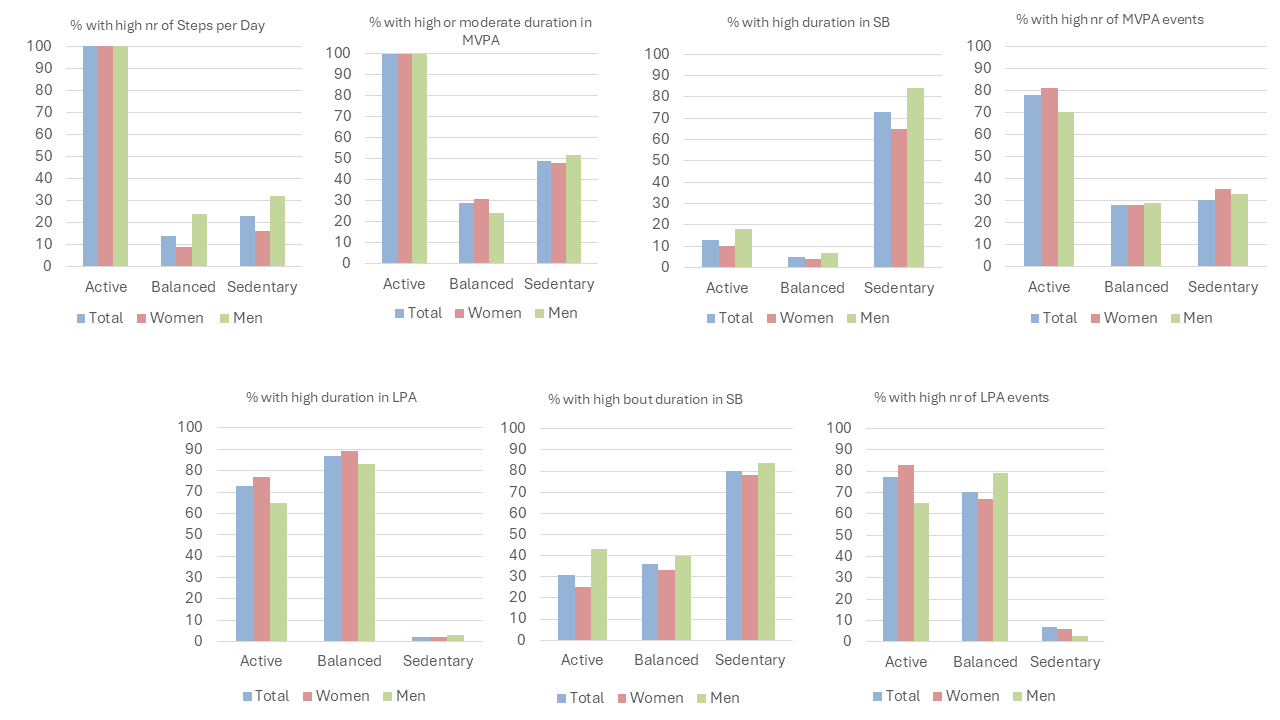


**Supplementary Table S4.** Prevalence of pain characteristics in each latent class of activity pattern

| Pain characteristics | Total sample  (n=665) | Active Movers  (n=282) | Balanced Movers  (n=138) | Sedentary Movers  (n=245) | Men  (n=239) | Women  (n=426) | Younger-old  (n=382) | Oldest-old  (n=283) |
| --- | --- | --- | --- | --- | --- | --- | --- | --- |
| Pain prevalence, n (%) |  |  |  |  |  |  |  |  |
| *Yes* | 230(34.6) | 81(28.7) | 49(35.5) | 100(40.8) | 61(25.5) | 169(39.7) | 121(31.7) | 109(38.5) |
| Pain intensity, n (%) |  |  |  |  |  |  |  |  |
| *Mild to Moderate* | 159(23.9) | 63(22.3) | 35(25.4) | 61(24.9) | 48(20.1) | 111(26.1) | 88(23.0) | 71(25.1) |
| *Moderately severe to Very severe* | 70(10.5) | 17(6.0) | 14(10.1) | 39(15.9) | 13(5.0) | 57(13.4) | 32(8.4) | 38(13.4) |
| Pain Frequency, n (%) |  |  |  |  |  |  |  |  |
| *Sometimes* | 93(14.0) | 38(13.5) | 21(15.2) | 34(13.9) | 31(13.0) | 62(14.6) | 54(14.1) | 39(13.8) |
| *Daily/almost daily* | 131(19.7) | 39(13.8) | 28(20.3) | 64(26.1) | 29(12.1) | 102(23.9) | 61(16.0) | 70(24.7) |
| Pain Locations, n (%) |  |  |  |  |  |  |  |  |
| *Single* | 116(17.4) | 42(14.9) | 24(17.4) | 50(20.4) | 39(16.3) | 77(18.1) | 71(18.6) | 45(15.9) |
| *Multiple* | 109(16.4) | 36(12.8) | 25(18.1) | 48(19.6) | 21(8.8) | 88(20.7) | 46(12.0) | 63(22.3) |
| Pain severity, n (%) |  |  |  |  |  |  |  |  |
| *Low* | 490(73.7) | 229(81.2) | 96(69.6) | 165(67.3) | 195(81.6) | 295(69.2) | 298(78.0) | 192(67.8) |
| *High* | 175(26.3) | 53(18.8) | 42(30.4) | 80(32.7) | 44(18.4) | 131(30.8) | 84(22.0) | 91(32.2) |

**Supplementary Table S5.** Interactions between pain characteristic*sex, and pain characteristics*age group (=66/ ≥80 years), Relative Risk Ratio (RRR) and 95% Confidence Intervals (CI)

|  |  |  | Interactions for pain * age group | |  | Interactions for pain * sex | |
| --- | --- | --- | --- | --- | --- | --- | --- |
| Pain characteristics | **Active Movers** |  | **Balanced Movers p-value** | **Sedentary movers**  **p-value** |  | **Balanced Movers p-value** | **Sedentary Movers p-value** |
| Pain previous 4 weeks |  |  |  |  |  |  |  |
| *Yes* | 1.0 |  | 0.586 | *0.036* |  | 0.933 | 0.160 |
| Pain intensity |  |  |  |  |  |  |  |
| *Mild to Moderate* | 1.0 |  | 0.322 | 0.120 |  | 0.834 | 0.737 |
| *Moderately severe to Very Severe* | 1.0 |  | 0.553 | 0.180 |  | 0.471 | *0.032* |
| Pain frequency |  |  |  |  |  |  |  |
| *Sometimes* | 1.0 |  | 0.642 | 0.822 |  | 0.593 | 0.803 |
| *Daily/almost daily* | 1.0 |  | 0.276 | *0.009* |  | 0.546 | 0.070 |
| Pain location |  |  |  |  |  |  |  |
| *Single* | 1.0 |  | 0.878 | 0.107 |  | 0.809 | 0.679 |
| *Multiple* | 1.0 |  | 0.493 | 0.122 |  | 0.673 | 0.100 |
| Pain Severity  *High* | 1.0 |  | 0.996 | 0.213 |  | 0.457 | 0.066 |

*Model 1; adjusted for sex, age, educational level.*

**Supplementary Table S6.** Relative Risk Ratio (RRR) and 95% Confidence Intervals (CI) across four latent classes of activity pattern, total sample

|  | Relative Risk Ratio (95% CI) from multinomial regression | | | | | | | | | | | | |
| --- | --- | --- | --- | --- | --- | --- | --- | --- | --- | --- | --- | --- | --- |
|  | **Model 1** | | | | | | | | **Model 2** | | | | |
| Pain characteristics | **PA class 1**  **(referent)** | **PA class 2** | | **PA class 3** | | | **PA class 4** | |  | **PA class 2** | | **PA class 3** | **PA class 4** |
| Pain  *Yes* | 1.0 | 1.32  (0.81, 2.15) | | 1.17  (0.68, 2.00) | | | **1.84**  **(1.20, 2.83)** | |  | 1.01  (0.59, 1.70) | | 1.07  (0.60, 1.91) | 1.41  (0.88, 2.27) |
| Pain intensity  *Mild to Moderate* | 1.0 | 1.31  (0.76, 2.26) | | 1.05  (0.57, 1.91) | | | 1.55  (0.96, 2.51) | |  | 1.09  (0.61, 1.95) | | 1.00  (0.52, 1.89) | 1.35  (0.80, 2.28) |
| *Moderately severe to Very severe* |  | 1.47  (0.64, 3.39) | | 1.67  (0.66, 4.21) | | | **3.05**  **(1.51, 6.17)** | |  | 0.88  (0.36, 2.13) | | 1.43  (0.55, 3.74) | 1.73  (0.80, 3.74) |
| Pain frequency |  |  |  | |  |  | |  | | |  |  |  |
| *Sometimes* | 1.0 | 1.27  (0.65, 2.48) | | 1.33  (0.66, 2.67) | | | 1.36  (0.75, 2.47) | |  | 1.03  (0.51, 2.07) | | 1.28  (0.63, 2.63) | 1.19  (0.63, 2.26) |
| *Daily/almost daily* | 1.0 | 1.49  (0.81, 2.75) | | 1.16  (0.56, 2.38) | | | **2.36**  **(1.39, 4.01)** | |  | 0.99  (0.51, 1.92) | | 0.93  (0.43, 2.01) | 1.56  (0.87, 2.80) |
| Pain Locations |  |  |  | |  |  | |  | | |  |  |  |
| *Single* | 1.0 | 1.53  (0.82, 2.84) | | 1.16  (0.59, 2.29) | | | **1.90**  **(1.10, 3.28)** | |  | 1.21  (0.63, 2.31) | | 1.01  (0.49, 2.05) | 1.59  (0.89, 2.85) |
| *Multiple* | 1.0 | 1.19  (0.62, 2.28) | | 1.13  (0.53, 2.37) | | | **1.76**  **(1.01, 3.09)** | |  | 0.78  (0.39, 1.58) | | 0.99  (0.45, 2.18) | 1.18  (0.63, 2.21) |
| Pain Severity  *High* | 1.0 | 1.58  (0.93, 2.68) | | 1.22  (0.67, 2.24) | | | **1.92**  **(1.20, 3.06)** | |  | 1.11  (0.63, 1.95) | | 1.03  (0.54, 1.97) | 1.32  (0.79, 2.21) |

*Model 1 adjusted for sex, age, educational level. Model 2 additionally adjusted for co-habitation status, number of chronic diseases, physical function, depressive symptoms. RRR= Relative Risk Ratio, CI= Confidence Interval, Bold = significance p ≤ 0.05*

**Supplementary Table S7.** Relative Risk Ratio (RRR) and 95% Confidence Intervals (CI) across four latent classes of activity pattern, with interactions for age category,

|  | Relative Risk Ratio (95% CI) from multinominal regression analysis | | | | | | | |
| --- | --- | --- | --- | --- | --- | --- | --- | --- |
|  | **Younger-old (= 66)**  **(n=376)** | | | | **Oldest-old (>80)**  **(n=281)** | | | |
| Pain characteristics | **Class 1**  **(referent)** | **Class 2** | **Class 3** | **Class 4** | **Class 1**  **(referent)** | **Class 2** | **Class 3** | **Class 4** |
| Pain previous 4 weeks |  |  |  |  |  |  |  |  |
| *Yes* | 1.0 | 0.92  (0.45, 1.88) | 0.79  (0.41, 1.51) | 0.89  (0.49, 1.61) | 1.0 | 1.38  (0.60, 3.17) | 1.50  (0.50, 4.50) | **2.19**  **(0.99, 4.81)** |
| Pain intensity |  |  |  |  |  |  |  |  |
| *Mild to Moderate* | 1.0 | 0.80  (0.35, 1.81) | 0.79  (0.38, 1.62) | 0.89  (0.45, 1.74) | 1.0 | 1.85  (0.72, 4.73) | 1.31  (0.37, 4.65) | 2.30  (0.93, 5.70) |
| *Moderately severe to Very severe* | 1.0 | 1.37  (0.45, 4.17) | 0.94  (0.30, 2.92) | 1.06  (0.39, 2.85) | 1.0 | 0.60  (0.15, 2.38) | 1.53  (0.30, 7.92) | 1.71  (0.50, 5.84) |
| Pain frequency |  | | | |  |  |  |  |
| *Sometimes* | 1.0 | 1.15  (0.51, 2.62) | 0.71  (0.31, 1.59) | 0.97  (0.47, 1.97) | 1.0 | 1.21  (0.40, 3.65) | 2.12  (0.59, 7.62) | 1.48  (0.51, 4.28) |
| *Daily/almost daily* | 1.0 | 0.59  (0.20, 1.78) | 0.76  (0.30, 1.88) | 0.77  (0.33, 1.80) | 1.0 | 1.48  (0.51, 4.34) | 0.70  (0.12, 3.97) | **2.77**  **(1.01, 7.62)** |
| Pain Locations |  | | | | | | | |
| *Single* | 1.0 | 1.36  (0.31, 6.02) | 0.27  (0.06, 1.18) | 1.52  (0.44, 5.30) | 1.0 | 2.05  (0.58, 7.25) | 2.36  (0.51, 10.90) | **3.72**  **(1.12, 12.34)** |
| *Multiple* | 1.0 | 0.79  (0.13, 4.67) | 0.78  (0.21, 2.88) | 1.11  (0.26, 4.74) | 1.0 | 1.02  (0.38, 2.70) | 0.97  (0.25, 3.75) | 1.48  (0.59, 3.73) |
| Pain Severity  *High* | 1.0 | 1.19  (0.56, 2.57) | 0.84  (0.40, 1.77) | 1.01  (0.52, 1.95) | 1.0 | 1.09  (0.46, 2.58) | 1.05  (0.33, 3.35) | 1.43  (0.63, 3.21) |

*All models adjusted for sex, age, educational level co-habitation status, number of chronic diseases, physical function, depressive symptoms. Interaction for pain characteristics*age group RRR= Relative Risk Ratio, CI= Confidence Interval.*

**Supplementary Table S8.** Risk pain characteristics across four latent classes of activity pattern, with interactions for sex, fully adjusted

|  | Relative Risk Ratio (95% CI) from multinominal regression analysis | | | | | | | |
| --- | --- | --- | --- | --- | --- | --- | --- | --- |
|  | **Men (n= 236)** | | | | **Women (n= 421)** | | | |
| Pain characteristics | **Class 1**  **(referent)** | **Class 2** | **Class 3** | **Class 4** | **Class 1**  **(referent)** | **Class 2** | **Class 3** | **Class 4** |
| Pain previous 4 weeks |  |  |  |  |  |  |  |  |
| *Yes* | 1.0 | 1.02  (0.39, 2.69) | 1.02  (0.40, 2.59) | 0.87  (0.37, 2.02) | 1.0 | 1.01  (0.55, 1.86) | 1.03  (0.50, 2.12) | **1.79**  **(1.01, 3.15)** |
| Pain intensity |  |  |  |  |  |  |  |  |
| *Mild to Moderate* | 1.0 | 1.32  (0.45, 3.88) | 1.17  (0.42, 3.25) | 1.20  (0.47, 3.04) | 1.0 | 1.04  (0.52, 2.04) | 0.88  (0.39, 2.02) | 1.51  (0.79, 2.87) |
| *Moderately severe to Very severe* | 1.0 | 0.34  (0.06, 2.02) | 0.54  (0.88, 3.27) | 0.23  (0.04, 1.19) | 1.0 | 1.08  (0.39, 2.96) | 1.73  (0.58, 5.21) | **2.87**  **(1.20, 6.87)** |
| Pain frequency |  | | | |  |  |  |  |
| *Sometimes* | 1.0 | 1.58  (0.45, 5.55) | 1.12  (0.31, 4.07) | 1.46  (0.48, 4.45) | 1.0 | 0.85  (0.36, 1.97) | 1.46  (0.62, 3.47) | 1.09  (0.49, 2.43) |
| *Daily/almost daily* | 1.0 | 0.61  (0.16, 2.31) | 0.88  (0.27, 2.86) | 0.48  (0.15, 1.50) | 1.0 | 1.16  (0.55, 2.44) | 0.79  (0.29, 2.17) | **2.30**  **(1.17, 4.53)** |
| Pain Locations |  | | | | | | | |
| *Single* | 1.0 | 1.32  (0.41, 4.26) | 1.15  (0.37, 3.55) | 1.20  (0.43, 3.36) | 1.0 | 1.17  (0.54, 2.56) | 0.87  (0.34, 2.24) | 1.93  (0.95, 3.93) |
| *Multiple* | 1.0 | 0.65  (0.15, 2.75) | 0.77  (0.19, 3.04) | 0.49  (0.14, 1.73) | 1.0 | 0.84  (0.38, 1.84) | 1.01  (0.40, 2.55) | 1.60  (0.78, 3.26) |
| Pain Severity  *High* | 1.0 | 0.80  (0.28, 2.32) | 1.01  (0.37, 2.76) | 0.54  (0.21, 1.39) | 1.0 | 1.27  (0.66, 2.44) | 0.90  (0.39, 2.04) | **1.93**  **(1.05, 3.53)** |

*All models adjusted for sex, age, educational level co-habitation status, number of chronic diseases, physical function, depressive symptoms. Interaction for pain characteristics*sex RRR= Relative Risk Ratio, CI= Confidence Interval, Bold = significance p <= 0.05*

**Supplementary Table S9.** Relative Risk Ratio (RRR) and 95% Confidence Intervals (CI) across latent classes of activity pattern—with Balanced Movers as referent category

|  | | Relative Risk Ratio (95% CI) from multinomial regression analysis | | | | | | | | | | | | | | | | | | | | |  |
| --- | --- | --- | --- | --- | --- | --- | --- | --- | --- | --- | --- | --- | --- | --- | --- | --- | --- | --- | --- | --- | --- | --- | --- |
|  | |  | | **Model 1** | | | | |  | | |  | | | | **Model 2** | | | | |  | |  |
| Pain characteristics | | **Balanced**  **Movers**  **(referent)** | **Active Movers** | | | **p-value** | | **Sedentary Movers** | | | **p-value** | | |  | **Active Movers** | | **p-value** | | **Sedentary Movers** | | | **p-value** | |
| Pain  *Yes* | | 1.0 | 0.74  (0.46, 1.17) | | | 0.198 | | 1.36  (0.87, 2.11) | | | 0.175 | | |  | 0.96  (0.58, 1.58) | | 0.874 | | 1.36  (0.85, 2.17) | | | 0.195 | |
| Pain intensity  *Mild to Moderate* | | 1.0 | 0.77  (0.46, 1.28) | | | 0.313 | | 1.12  (0.68, 1.85) | | | 0.655 | | |  | 0.93  (0.54, 1.61) | | 0.790 | | 1.15  (0.68, 1.94) | | | 0.606 | |
| *Moderately severe to Very severe* |  | 0.59  (0.26, 1.30) | | | 0.188 | | **2.01**  **(1.02, 3.97)** | | | *0.043* | | |  | 0.90  (0.39, 2.10) | | 0.816 | | 1.95  (0.96, 3.97) | | | 0.064 | |  |
| Pain frequency | |  |  | |  | |  | | |  | | |  | |  | | |  | |  |  |  |  |
| *Sometimes* | | 1.0 | 0.73  (0.39, 1.36) | | | 0.321 | | 0.99  (0.54, 1.82) | | | 0.981 | | |  | 0.88  (0.46, 1.69) | | 0.709 | | 1.09  (0.58, 2.05) | | | 0.785 | |
| *Daily/almost daily* | | 1.0 | 0.68  (0.38, 1.23) | | | 0.204 | | 1.58  (0.93, 2.69) | | | 0.089 | | |  | 1.01  (0.54, 1.90) | | 0.978 | | 1.54  (0.87, 2.71) | | | 0.136 | |
| Pain Locations | |  |  | |  | |  | | |  | | |  | |  | | |  | |  |  |  |  |
| *Single* | | 1.0 | 0.66  (0.36, 1.20) | | | 0.171 | | 1.27  (0.73, 2.22) | | | 0.401 | | |  | 0.83  (0.44, 1.53) | | 0.545 | | 1.33  (0.74, 2.37) | | | 0.340 | |
| *Multiple* | | 1.0 | 0.76  (0.41, 1.40) | | | 0.378 | | 1.34  (0.76, 2.37) | | | 0.304 | | |  | 1.10  (0.57, 2.14) | | 0.768 | | 1.36  (0.75, 2.47) | | | 0.312 | |
| Pain Severity  *High* | | 1.0 | **0.57**  **(0.37, 0.95)** | | | 0.029 | | 1.22  (0.77, 1.93) | | | 0.405 | | |  | 0.79  (0.46, 1.36) | | 0.400 | | 1.19  (0.73, 1.95) | | | 0.475 | |

*Model 1 adjusted for age, sex and educational level. Model 2 additionally adjusted for co-habitation status, number of chronic diseases, physical function, depressive symptoms. RRR= Relative Risk Ratio, CI= Confidence Interval.*

**Supplementary Table S10.** Relative Risk Ratio (RRR) and 95% Confidence Intervals (CI) across latent classes of activity pattern, with interactions for sex—with Balanced Movers as referent category. Interaction for pain characteristics*sex

|  | Relative Risk Ratio (95% CI) from multinominal regression analysis | | | | | | | | | | |
| --- | --- | --- | --- | --- | --- | --- | --- | --- | --- | --- | --- |
|  |  |  | **Younger-old (n=382)**  *Balanced Movers (n=50)* | | **Oldest- old (n= 283)**  *Balanced Movers (n=88)* | |  | **Men (n=239)**  *Balanced Movers (n=42)* | | **Women (n=426)**  *Balanced Movers (n=96)* | |
|  | **Balanced movers**  (referent) |  | **Active Movers**  (n=219) | **Sedentary Movers**  (n=113) | **Active Movers**  (n=63) | **Sedentary Movers**  (n=132) |  | **Active Movers**  (n=93) | **Sedentary Movers**  (n=104) | **Active Movers**  (n=189) | **Sedentary Movers**  (n=141) |
| Pain previous 4 weeks |  |  |  |  |  |  |  |  |  |  |  |
| *Yes* | 1.0 |  | 1.00  (0.50, 1.98) | 0.89  (0.43, 1.83) | 0.71  (0.33, 1.54) | 1.61  (0.89, 2.91) |  | 0.98  (0.40, 2.43) | 0.92  (0.40, 2.13) | 0.94  (0.52, 1.68) | 1.64  (0.94, 2.85) |
| Pain intensity |  |  |  |  |  |  |  |  |  |  |  |
| *Mild to Moderate* | 1.0 |  | 1.26  (0.57, 2.80) | 1.07  (0.46, 2.48) | 0.56  (0.24, 1.33) | 1.15  (0.59, 2.25) |  | 0.87  (0.33, 2.34) | 0.92  (0.37, 2.30) | 0.94  (0.49, 1.80) | 1.30  (0.69, 2.45) |
| *Moderately severe to Very severe* | 1.0 |  | 0.56  (0.20, 1.59) | 0.67  (0.24, 1.92) | 1.23  (0.31, 4.85) | **3.16**  **(1.24, 8.06)** |  | 1.62  (0.29, 9.22) | 0.95  (0.21, 4.27) | 0.79  (0.31, 2.04) | **2.42**  **(1.09, 5.37)** |
| Pain frequency |  |  |  |  |  |  |  |  |  |  |  |
| *Sometimes* | 1.0 |  | 0.85  (0.35, 2.06) | 0.87  (0.34, 2.19) | 0.86  (0.32, 2.32) | 1.23  (0.52, 2.87) |  | 0.69  (0.22, 2.19) | 0.94  (0.34, 2.59) | 0.97  (0.44, 2.14) | 1.19  (0.53, 2.63) |
| *Daily/almost daily* | 1.0 |  | 1.11  (0.46, 2.64) | 0.86  (0.34, 2.15) | 0.54  (0.19, 1.56) | 1.85  (0.93, 3.69) |  | 1.47  (0.42, 5.12) | 0.93  (0.29, 2.98) | 0.87  (0.42, 1.78) | 1.84  (0.97, 3.48) |
| Pain locations |  |  |  |  |  |  |  |  |  |  |  |
| *Single* | 1.0 |  | 0.82  (0.37, 1.81) | 0.83  (0.36, 1.89) | 0.58  (0.19, 1.78) | 1.89  (0.85, 4.19) |  | 0.72  (0.24, 2.14) | 1.01  (0.39, 2.63) | 0.87  (0.41, 1.83) | 1.56  (0.76, 3.22) |
| *Multiple* | 1.0 |  | 1.33  (0.48, 3.65) | 1.03  (0.36, 2.98) | 0.82  (0.32, 2.08) | 1.39  (0.69, 2.81) |  | 1.61  (0.42, 6.20) | 0.78  (0.21, 2.88) | 0.96  (0.45, 2.02) | 1.65  (0.85, 3.23) |
| Pain Severity  *High* | 1.0 |  | 0.73  (0.35, 1.52) | 0.80  (0.38, 1.70) | 0.74  (0.33, 1.68) | 1.37  (0.74, 2.52) |  | 1.11  (0.41, 3.02) | 0.83  (0.33, 2.09) | 0.68  (0.37, 1.28) | 1.41  (0.80, 2.49) |

*Adjusted for sex, age, educational level, co-habitation status, number of chronic diseases, physical function. Interaction for pain characteristics*sex RRR= Relative Risk Ratio, CI= Confidence Interval, RRR= Relative Risk Ratio, CI= Confidence Interval. Bold = significance, p ≤0.05*

**Supplementary Table S11.** Relative Risk Ratio (RRR) and 95% Confidence Intervals (CI) across latent classes of activity pattern, with interactions for musculoskeletal disorder (yes/no) and analgesic drugs (user/non-user)

|  | Relative Risk Ratio (95% CI) from multinominal regression analysis | | | | | | | | | | |
| --- | --- | --- | --- | --- | --- | --- | --- | --- | --- | --- | --- |
|  |  |  | **Musculoskeletal disorder, yes (n= 391)** | | **Musculoskeletal disorder, no (n=274)** | |  | **User of analgesic drugs (n=229)** | | **Non-user of analgesic drugs (n=436)** | |
|  | **Active Movers**  (referent) |  | **Balanced Movers**  (n=89) | **Sedentary Movers**  (n=163) | **Balanced Movers**  (n=49) | **Sedentary Movers**  (n=82) |  | **Balanced Movers**  (n=49) | **Sedentary Movers**  (n=98) | **Balanced Movers**  (n=89) | **Sedentary Movers**  (n=147) |
| Pain previous 4 weeks |  |  |  |  |  |  |  |  |  |  |  |
| *Yes* | 1.0 |  | 0.88  (0.47, 1.63) | 1.34  (0.78, 2.30) | 1.65  (0.70, 3.88) | 1.53  (0.72, 3.26) |  | 1.10  (0.49, 2.47) | 1.45  (0.73, 2.88) | 1.03  (0.52, 2.03) | 1.16  (0.64, 2.07) |
| Pain intensity |  |  |  |  |  |  |  |  |  |  |  |
| *Mild to Moderate* | 1.0 |  | 0.80  (0.40, 1.60) | 1.13  (0.62, 2.03) | 2.27  (0.92, 5.64) | 1.43  (0.60, 3.40) |  | 1.44  (0.60, 3.47) | 1.21  (0.55, 2.64) | 0.83  (0.40, 1.75) | 1.17  (0.64, 2.14) |
| *Moderately severe to Very severe* | 1.0 |  | 1.33  (0.51, 3.49) | **2.36**  **(1.02, 5.49)** | 0.36  (0.04, 3.55) | 1.59  (0.40, 6.35) |  | 0.79  (0.26, 2.43) | 2.06  (0.86, 4.97) | 2.74  (0.56, 13.38) | 1.36  (0.28, 6.67) |
| Pain frequency |  |  |  |  |  |  |  |  |  |  |  |
| *Sometimes* | 1.0 |  | 0.97  (0.43, 2.17) | 1.05  (0.51, 2.15) | 1.64  (0.51, 5.22) | 1.77  (0.65, 4.77) |  | 1.85  (0.65, 5.31) | 1.49  (0.58, 3.85) | 0.80  (0.34, 1.88) | 0.99  (0.49, 2.03) |
| *Daily/almost daily* | 1.0 |  | 0.84  (0.40, 1.78) | 1.56  (0.83, 2.93) | 1.79  (0.56, 5.73) | 1.16  (0.38, 3.52) |  | 0.87  (0.35, 2.16) | 1.45  (0.68, 3.07) | 1.48  (0.56, 3.92) | 1.36  (0.57, 3.26) |
| Pain locations |  |  |  |  |  |  |  |  |  |  |  |
| *Single* | 1.0 |  | 0.89  (0.40, 1.96) | 1.33  (0.68, 2.62) | 2.19  (0.80, 6.04) | 2.17  (0.91, 5.19) |  | 1.43  (0.54, 3.79) | 1.74  (0.77, 3.95) | 1.08  (0.46, 2.51) | 1.36  (0.67, 2.77) |
| *Multiple* | 1.0 |  | 0.89  (0.42, 1.91) | 1.35  (0.70, 2.61) | 0.87  (0.21, 3.50) | 0.61  (0.15, 2.47) |  | 0.96  (0.37, 2.50) | 1.27  (0.56, 2.87) | 0.95  (0.36, 2.48) | 0.90  (0.39, 2.11) |
| Pain Severity  *High* | 1.0 |  | 1.28  (0.68, 2.42) | 1.66  (0.65, 2.09) | 1.47  (0.53, 4.08) | 1.11  (0.43, 2.86) |  | 0.97  (0.43, 2.14) | 1.34  (0.68, 2.63) | 1.81  (0.82, 3.98) | 1.40  (0.68, 2.88) |

*Adjusted for sex, age, educational level, co-habitation status, number of chronic diseases, physical function and depressive symptoms. Interaction for pain characteristics*musculoskeletal disorder, pain characteristics*use of analgesic drug RRR= Relative Risk Ratio, CI= Confidence Interval, RRR= Relative Risk Ratio, CI= Confidence Interval. Bold = significance, p ≤0.05*
